# Cardiopulmonary parameters in patients with Tetralogy of Fallot: the reference values for treadmill and cycle ergometer

**DOI:** 10.3389/fcvm.2026.1673478

**Published:** 2026-04-21

**Authors:** Benedetta Leonardi, Federica Gentili, Eliana Tranchita, Claudia Montanaro, Gianfranco Butera, Rosalinda Palmieri, Giulia Di Già, Aurelio Secinaro, Giovanni Antonelli, Michele Lioncino, Ugo Giordano

**Affiliations:** Bambino Gesù Children's Hospital, IRCCS, Rome, Italy

**Keywords:** cardiopulmonary test, cyclo-ergometer, Tetralogy of Fallot, treadmil, VE/VCO₂ slope, VO2 peak

## Abstract

**Background:**

The aim of this study was to establish sex- and age-specific reference values for ramp cycle-ergometer and treadmill cardiopulmonary exercise testing (CPET) in patients with Tetralogy of Fallot (ToF). Despite successful surgical repair, residual pulmonary regurgitation remains common in repaired ToF (rToF), often leading to right or left ventricular dysfunction and reduced exercise capacity. CPET is a reliable tool for evaluating cardiopulmonary function. Although both treadmill and cycle ergometer protocols are used interchangeably, reference value ranges for each method in this population remain unclear.

**Method:**

CPET data were collected from asymptomatic rToF patients who had undergone cardiac magnetic resonance imaging (CMR) and performed CPET on a treadmill or cycle ergometer between 2020 and 2024. Physical activity was assessed using the International Physical Activity Questionnaire (IPAQ).

**Results:**

Among 290 patients, median age at CPET was 21.6 years (15.9–29.3), and median BMI was 22.5 kg/m^2^ (20.0–24.9). Nearly all participants (99%) were in NYHA class I. Physical activity was classified as sedentary in 29%, moderate in 61%, and high in 9%. Median VO₂ peak was 26.4 mL/min/kg (23.0–31.5), corresponding to 72.9% predicted (62.4–83.2). Median oxygen pulse at peak was 9.9 mL/beat (8.2–12.0), with 76.9% predicted (68.0–87.5). Median VE/VCO₂ slope at the respiratory compensation point was 29.0 (26.0–32.6), and median oxygen uptake efficiency slope (OUES) was 1,792.5 mL/min/log(L/min) (1,535.0–2,181.5). VO₂ peak and percent-predicted VO₂ were significantly higher with treadmill testing in both sexes (*p* < 0.05); oxygen pulse was higher only in females. VE/VCO₂ slope and OUES were unaffected by modality. When stratified by age (<18 vs. ≥18 years), treadmill-related differences in VO₂ peak and percent-predicted VO₂ remained significant in both sexes ≥18 years, and in females <18 years. Oxygen pulse was significantly higher with treadmill only in females ≥18 years. VE/VCO₂ slope and OUES remained unchanged across modalities and age groups

**Conclusion:**

This study provides CPET values stratified by modality and sex in a large cohort of asymptomatic rToF patients, offering valuable reference data for clinical assessment. Future studies should validate pediatric normative CPET values through prospective, inclusive, statistically powered cohorts using standardized protocols and cross-center comparability.

## 1 Background

Surgical correction of Tetralogy of Fallot (ToF) in early childhood frequently results in chronic pulmonary insufficiency. This complication can lead, over time, to right ventricular (RV) dilatation and dysfunction, left ventricular (LV) dysfunction, and potentially life-threatening ventricular arrhythmias (1, 2). Although most patients report being asymptomatic, chronic RV volume overload may still impair functional capacity. Cardiopulmonary exercise testing (CPET) is a well-established tool for assessing cardiovascular reserve—closely linked to cardiac output—via oxygen consumption (VO₂) in both healthy individuals and those with cardiovascular disease (3). Accordingly, CPET has well-established prognostic value in various cardiac conditions and has also proven effective in assessing the true clinical status of patients with repaired ToF (rToF). Key prognostic indicators—including peak oxygen consumption (VO₂ peak), the ventilatory efficiency slope (VE/VCO₂ slope), and heart rate recovery (HRR)—are crucial for risk stratification in this population. The clinical utility and strategic importance of CPET in the long-term follow-up of rToF patients have been consistently highlighted in the literature (3–6). Despite the well-established prognostic role of CPET in rToF, important limitations persist in the existing literature regarding the optimal use and interpretation of this test in congenital heart disease. irst, most available CPET reference values are derived from healthy populations. In the relatively few studies focusing on congenital heart disease, daily physical activity levels and cardiac magnetic resonance imaging data have rarely been considered simultaneously, limiting the clinical interpretability of CPET results within the complex pathophysiology of repaired congenital defects. Second, prior studies have typically employed a single exercise modality—most commonly cycle ergometry—without systematically accounting for the potential influence of exercise modality on CPET-derived parameters. As a result, although modality-related differences in VO₂ peak are well documented in healthy individuals and in patients with heart failure, the impact of exercise modality on CPET parameters in rToF remains insufficiently recognized, and modality-specific reference values are largely lacking. This represents a relevant gap, particularly given the increasing reliance on CPET-derived indices for longitudinal assessment and clinical decision-making in this population. Notably, current guidelines for adults with congenital heart disease do not indicate a preferred exercise modality for CPET. Existing recommendations primarily address safety considerations, with the American Heart Association reporting a very low risk of serious adverse events during CPET (<0.01% with cycle ergometry and <0.2% with treadmill testing). However, large-scale studies directly comparing the safety and physiological responses associated with different CPET modalities in congenital heart disease populations are still lacking. Consequently, no specific guidance is available regarding the most appropriate exercise modality for the wide spectrum of congenital anatomical defects and their heterogeneous clinical and imaging presentations. Evidence from acquired heart disease consistently demonstrates higher VO₂ peak values during treadmill testing compared with cycle ergometry. Studies in patients with heart failure and large registries such as the FRIEND database have shown that treadmill-derived VO₂ peak values are, on average, 15%–25% higher across age and sex groups, while ventilatory efficiency remains largely comparable between modalities (7–9). Maeder et al. (7) demonstrated that VO₂ peak was significantly higher during treadmill testing compared to bicycle testing in adults with mild heart failure. Likewise, Mazaheri et al. reported higher VO₂ peak values on the treadmill in patients with heart failure and severely reduced ejection fraction. Additionally, a large-scale study involving 15,045 CPETs in adults with a history of coronary artery bypass grafting, myocardial infarction, percutaneous coronary intervention, or heart failure found significant differences in VO₂ peak across sex and age groups (*P* < 0.001) (9). On average, men exhibited VO₂ peak values 23% higher than women, and a decline of approximately 7% per decade was observed in both sexes. Notably, treadmill testing yielded VO₂ peak values that were, on average, 21% higher than those obtained with cycle ergometry across all age groups. These differences were consistent across various categories of cardiovascular disease, as shown in the FRIEND registry (9). In Kaminsky et al. (10) (50th percentile) values for each decade were lower by 2.4 to 4.6 mL O₂·kg^−1^·min^−1^ and 1.5 to 3.8 mL O₂·kg^−1^·min^−1^, respectively. In women, the updated values were also reduced, though the differences were much smaller across all age groups, with declines in mean and median values ranging from 0.4 to 1.4 and 0.4 to 1.9 mL O₂·kg^−1^·min^−1^, respectively.Interestingly, no significant differences in VE/VCO₂ slope values were observed between the two exercise modalities (7). In contrast, whether and to what extent these modality-related differences apply to the complex physiology of repaired Tetralogy of Fallot has remained largely unexplored. In particular, there is limited discussion in the literature regarding the potential advantages of treadmill testing in rToF patients, who frequently develop chronotropic incompetence with advancing age and in whom achieving an adequate heart rate response is clinically relevant. n a previous study, we demonstrated that, in the same patients with repaired Tetralogy of Fallot, CPET performed on a treadmill vs. a cycle ergometer resulted in significant differences in specific cardiopulmonary parameters, with treadmill testing yielding VO₂ peak values consistently 10%–20% higher, while VE/VCO₂ slope remained comparable between modalities (11), like some previous studies in different population (7, 8, 12). These findings highlight the need for reference values that explicitly account for exercise modality. Moreover, sex, age, body mass index, and physical activity level are all known to contribute to variability in CPET outcomes, with females typically exhibiting lower VO₂ peak values normalized to body mass and higher resting heart rates, particularly among physically trained individuals (13). Taken together, the absence of disease-specific physiology and exercise modality reference values limits the accurate clinical interpretation of CPET in rToF patients. This issue is particularly relevant given the frequent use of CPET-derived indices in the longitudinal follow-up of this population, including decision-making related to pulmonary valve replacement. Accordingly, the present study aims to define modality-specific reference values for key CPET parameters in a large cohort of patients with rToF.

## 2 Materials and methods

The study had a retrospective design. Inclusion criteria were a diagnosis of repaired Tetralogy of Fallot (rToF), regular follow-up at our institution, age >12 years, and asymptomatic clinical status. Exclusion criteria included New York Heart Association (NYHA) functional class ≥ III–IV and right ventricular (RV) systolic pressure ≥ two-thirds of systemic pressure, as assessed by echocardiography. Pregnant patients and individuals with obesity (body mass index >30 kg/m^2^) were also excluded. The research protocol included a complete clinical evaluation, an international physical activity questionnaire (IPAQ), a 12-lead electrocardiogram (excluding the possibility of an arrhythmia), echocardiogram and cardiac MRI. The study was approved by the Ethics Committee of the Bambino Gesù Children's Hospital, IRCCS (Prot. Number 341/2015), and all subjects signed an informed consent form. The study was conducted in accordance with the Declaration of Helsinki.

### 2.1 Cardiac magnetic resonance assessment

Cardiac MRI exams were conducted on a 1.5 T scanner (AERA, Siemens, Erlangen, Germany), following a standardized protocol for repaired Tetralogy of Fallot (rToF) patients described in the literature. The imaging protocol included several sequences to assess cardiac anatomy, cine steady-state free precession for ventricular volume and function, and phase-contrast imaging to measure flow across the pulmonary valve, aortic valve, and both pulmonary arteries.

### 2.2 Cardiopulmonary exercise test assessment

Before each CPET, spirometry was performed, and patients' height and weight were recorded to calculate BSA and body mass index (BMI). Breath-by-breath gas exchange was monitored according to international guidelines. A 12-lead ECG was continuously recorded, while blood pressure and pulse oximetry were measured at 2- to 3-minute intervals and at peak exercise.

The standard incremental Bruce protocol was applied during the treadmill CPET, whereas the cycle ergometer CPET (Cosmed Quark PFT Cycle ergometer Technogym bike 1000 Med) was performed using a ramp protocol, with individualized workload increments calculated by the Wassermann equation, to be completed in a time range between 8 and 12 min; the patients were asked to keep a constant pace of 65–70 revolutions per minute (rpm). All patients were strongly verbally encouraged throughout the test to maintain the cadence of ±5 rpm and to achieve maximal effort. In both tests, patients exercised to volitional fatigue or until the occurrence of symptoms and/or appearance of threatening arrhythmias (supraventricular or ventricular tachycardia, atrial fibrillation). Tests were considered maximal when at least two of the following criteria were achieved: 1) failure to maintain the work rate, 2) respiratory exchange ratio (RER) > 1.1) maximal HR > 85% of age-predicted maximum (220-age), 4) occurrence of a VO2 plateau (VO2 increase ≤150 mL/min over the last 30s of the test). VO2 peak was calculated in both tests as the 15-seconds average of the highest VO2 achieved during the test. For the CPET data, percentages of the predicted values of VO2 peak were determined using Burstein et al. reference values for ramp cycle ergometer tests in patients younger than 18 years old. Wasserman equations were used for both tests in those older than 18 years old. The percentage of 80% of VO2 peak was considered the threshold of normality. The following maximal cardiopulmonary exercise parameters were collected and analysed: peak oxygen uptake normalized to body weight (peak VO₂/kg), percent-predicted oxygen uptake, the ventilatory equivalent for carbon dioxide (VE/VCO₂ slope) measured up to the respiratory compensation point (RCP), percent-predicted oxygen pulse (defined as the ratio of oxygen uptake to heart rate), and oxygen uptake efficiency slope (OUES).

#### 2.2.1 Physical activity questionnaire (IPAQ)

Physical activity level was assessed using the short form of the International Physical Activity Questionnaire (IPAQ), which estimates weekly energy expenditure expressed in MET-minutes, where MET (Metabolic Equivalent of Task) represents the energy cost of physical activities relative to the resting metabolic rate (1 MET corresponding to an oxygen consumption of 3.5 mL/kg/min), and based on the total score participants were classified as inactive if total weekly energy expenditure was below 700 MET-min, sufficiently active if between 700 and 2,519 MET-min, and active or very active if equal to or above 2,520 MET-min.

### 2.3 Statistical analysis

The descriptive statistics were expressed as mean with standard deviations or median with interquartile range (IQR) for continuous variables and as counts and percentages for categorical variables. Normality was assessed using the Shapiro–Wilk test. For the comparison of normally distributed continuous variables, the independent samples t-test was used, and in case of skewed distribution, the Mann–Whitney U-test or the Krustall-Wallis test was applied, as appropriate. Multivariable linear regression models were constructed for each cardiopulmonary exercise testing (CPET) parameter of interest (e.g., peak VO₂, % predicted VO₂, peak O₂ pulse, VE/VCO₂ slope, OUES). The primary independent variable was exercise modality (treadmill vs. cycle ergometer). Covariates included sex and age at the time of CPET for parameters expressed in absolute values. This approach was adopted to determine whether differences in CPET parameters between treadmill and bicycle testing persisted after adjusting for potential confounders. Regression coefficients (*β*), 95% confidence intervals (CI), and *p*-values were reported for each model. All statistical analyses were performed using R.

## 3 Results

A total of 290 patients with Tetralogy of Fallot (128 females and 162 males) who had undergone corrective surgery during childhood were included in the present study. Most patients were in NYHA functional class I. At the time of cardiopulmonary exercise testing (CPET), the median age at the time of cardiopulmonary exercise testing was 21.6 (15.9–29.3) years, and most patients (61%) were classified as sufficiently active. The most common type of surgical repair was transannular patch repair (79%).

The median peak oxygen consumption (VO₂ peak) was 26.4 mL/min/kg (23.0–31.5), corresponding to a median predicted percentage according to Wasserman of 72.9% (62.4–83.2). The median oxygen pulse at peak exercise was 9.9 mL/beat (8.2–12.0), with a median predicted percentage of 76.9% (68.0–87.5). The VE/VCO₂ slope at the respiratory compensation point showed a median value of 29.0 (26.0–32.6), while the median oxygen uptake efficiency slope (OUES) was 1,792.5 mL/min/log(L/min) (1,535.0–2,181.5). No episodes of ventricular arrhythmia occurred during CPET. Mean and median values of demographic, CPET, and cardiac MRI variables are presented in Table 1 according to sex.

**Table 1:** This table summarizes demographic, clinical, CPET, and CMR characteristics of the study population, stratified by sex (female and male).

Population characteristics	F *N* = 128^a^	M *N* = 162^a^
NYHA		
1	126 (98%)	161 (99%)
2	2 (1.6%)	1 (0.6%)
Bmi (kg/m2)		
Mean (SD)	22.4 (3.9)	23.3 (4.5)
Median (Q1, Q3)	22.3 (19.8, 24.3)	23.1 (20.3, 25.8)
IPAQ		
0	22 (35%)	13 (23%)
1	34 (55%)	39 (68%)
2	6 (9.7%)	5 (8.8%)
Age at CPET		
Mean (SD)	23.5 (8.4)	22.7 (8.2)
Median (Q1, Q3)	21.3 (16.5, 30.2)	21.6 (15.2, 28.7)
VO2 peak (kg/mL/min)		
Mean (SD)	25.6 (5.9)	28.9 (7.1)
Median (Q1, Q3)	24.8 (21.9, 28.9)	28.4 (24.4, 33.0)
VO2 pred (%)		
Mean (SD)	80.9 (15.1)	68.6 (14.3)
Median (Q1, Q3)	80.1 (70.1, 91.0)	67.4 (58.6, 77.6)
Peak O₂ pulse (mL/beat)		
Mean (SD)	8.7 (1.9)	11.5 (2.8)
Median (Q1, Q3)	8.6 (7.3, 9.8)	11.4 (9.5, 13.2)
Peak O₂ pulse pred (%)		
Mean (SD)	83.0 (13.9)	74.5 (15.3)
Median (Q1, Q3)	81.8 (74.0, 93.0)	72.9 (64.0, 84.7)
VE/VCO₂ slope at RCP		
Mean (SD)	30.2 (4.6)	29.0 (4.8)
Median (Q1, Q3)	29.2 (26.9, 33.0)	28.9 (25.4, 32.2)
Oues (mL/min/min)		
Mean (SD)	1,608.8 (375.8)	2,134.1 (582.7)
Median (Q1, Q3)	1,582.0 (1,352.0, 1,820.0)	2,056.0 (1,678.0, 2,502.0)
rvedvi (mL/m2)		
Mean (SD)	115.4 (24.0)	123.1 (24.6)
Median (Q1, Q3)	113.1 (100.6, 126.8)	122.1 (108.2, 137.7)
rvesvi (mL/m2)		
Mean (SD)	51.8 (15.4)	59.4 (15.5)
Median (Q1, Q3)	51.1 (40.8, 59.6)	58.1 (48.1, 67.3)
rvef (%)		
Mean (SD)	55.4 (6.2)	52.3 (8.1)
Median (Q1, Q3)	55.5 (50.4, 60.0)	51.4 (48.7, 55.8)
lvef (%)		
Mean (SD)	58.9 (6.5)	56.8 (7.6)
Median (Q1, Q3)	58.9 (55.9, 62.7)	56.0 (53.4, 59.0)
lvedvi (mL/m2)		
Mean (SD)	78.8 (14.6)	85.6 (16.1)
Median (Q1, Q3)	75.4 (69.9, 86.5)	85.8 (74.0, 95.1)
lvesvi (mL/m2)		
Mean (SD)	32.4 (7.9)	37.7 (9.5)
Median (Q1, Q3)	31.2 (27.4, 36.5)	38.0 (31.4, 42.9)
Pr (%)		
Mean (SD)	24.6 (17.7)	21.6 (17.2)
Median (Q1, Q3)	26.0 (5.9, 39.0)	19.3 (5.3, 36.0)

IPAQ, International physical activity questionnaire; LVEDVI, left ventricular end-diastolic volume indexed to body surface area; LVESVI, left ventricular end-systolic volume indexed to body surface area; LVEF, Left ventricle ejection fraction; RVEF, Right ventricle ejection fraction; PR, Pulmonary regurgitation; RVEDVI, right ventricular end-diastolic volume indexed to body surface area; RVESVI, Right ventricular end-systolic volume indexed to body surface area; OUES, Oxygen uptake efficiency slope.

^a^ *n* (%).

Spirometry values, obtained prior to exercise, were within normal limits and did not differ significantly between modalities, selected age groups, or sex.

**Figure 1:**
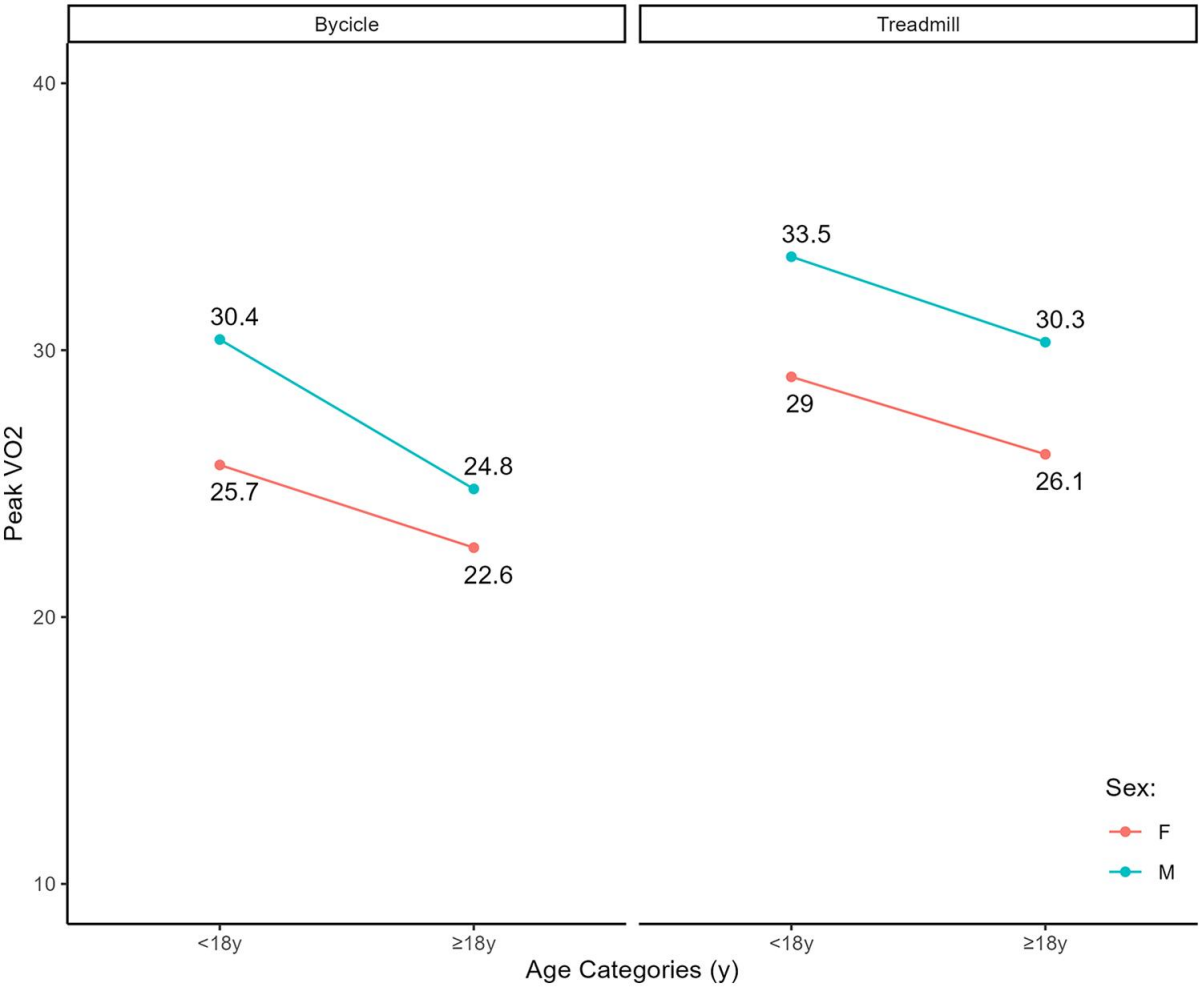
Distribution of VO₂ values by age group and exercise modality, separately for males and females.

**Figure 2:**
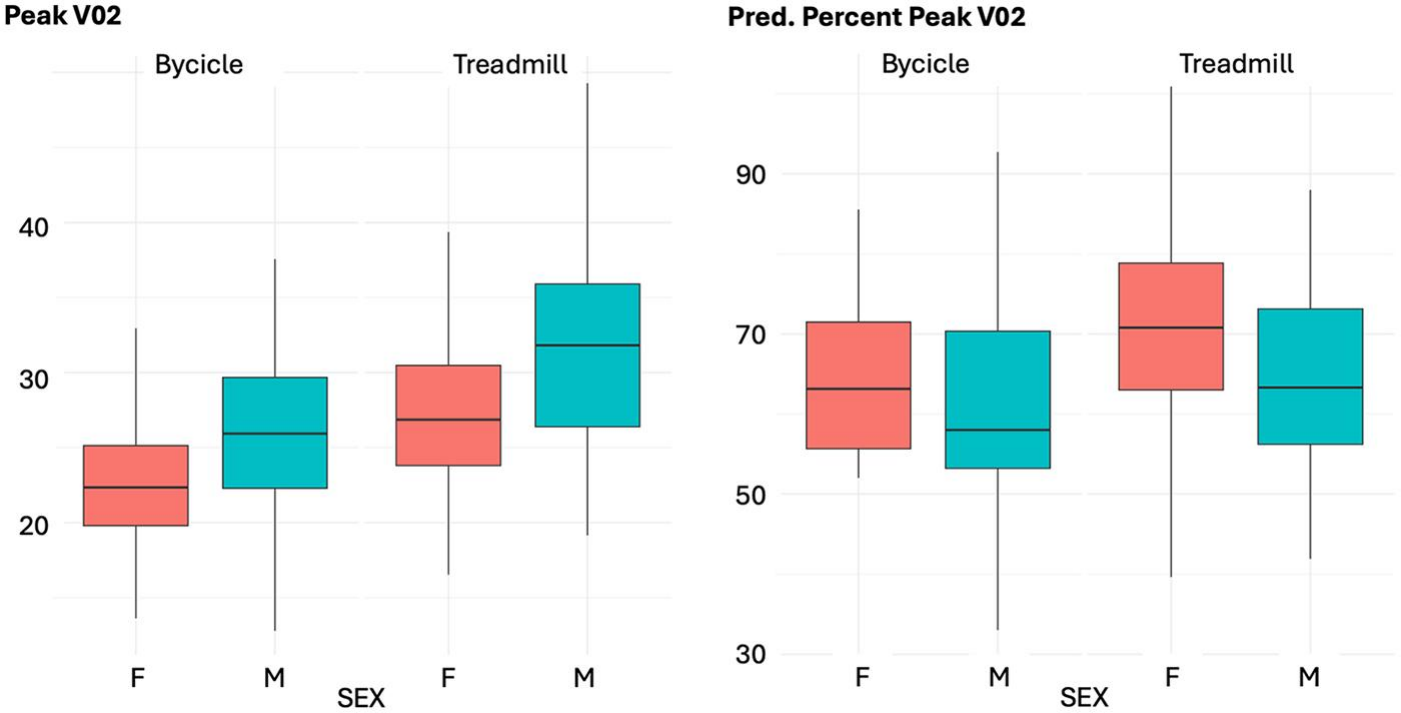
Comparison between treadmill and bicycle tests for peak VO₂ and its percent-predicted value. Scatter plots show the distribution of measured (left panel) and percent-predicted (right panel) peak oxygen consumption according to sex and exercise modality.

**Figure 3:**
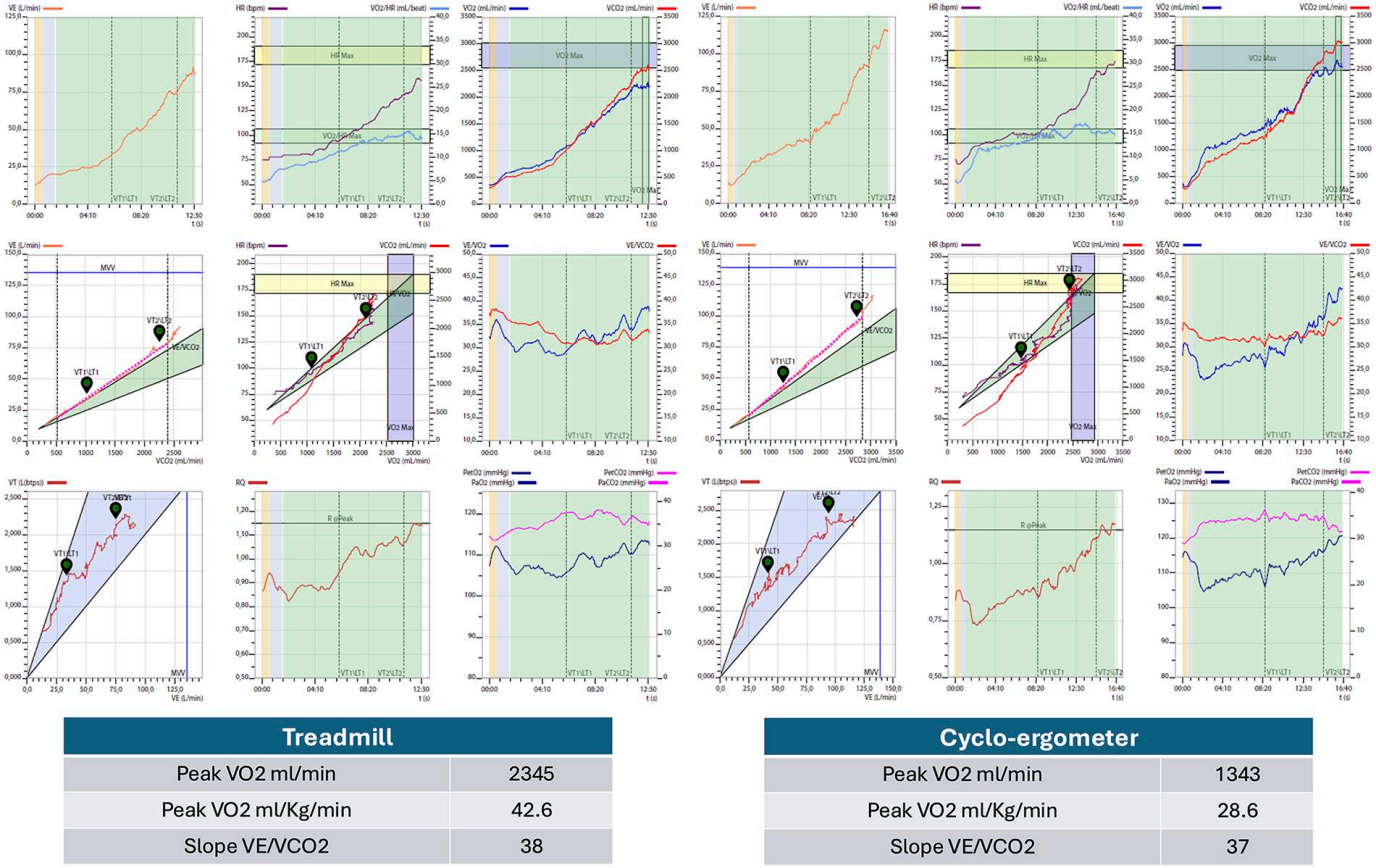
Example of comparison between the two methods for the following CPET parameters: peak oxygen consumption, predicted peak oxygen consumption, and VE/VCO₂ slope.

A detailed comparison of cardiopulmonary and MRI parameters between the two exercise modalities is provided by sex in Table 2. When stratifying the cohort by sex and test modality, no significant differences were observed in age at CPET, BMI, or IPAQ scores between subgroups (Table 2). VO₂ peak and its percent-predicted value were significantly higher with treadmill testing compared to cycle ergometry (Figures 1–3), with consistently higher baseline values observed in males (Figure 1). Interestingly, the difference between modalities reached statistical significance for oxygen pulse and its percent-predicted value only in females, with higher values recorded during treadmill testing. VE/VCO₂ slope and OUES were not influenced by the type of exercise modality. The regression analysis, performed to determine whether treadmill exercise yielded systematically different VO₂ peak values compared with cycle ergometer testing after adjusting for age and sex, showed that treadmill testing was significantly associated with higher absolute VO₂ peak (*β* = 4.2, 95% CI 2.8–5.6, *p* < 0.001), higher percent predicted VO₂ peak (*β* = 4.7, 95% CI 1.2–8.3, *p* = 0.010), and higher oxygen pulse (*β* = 0.78, 95% CI 0.20–1.4, *p* = 0.008) compared with bicycle testing. No significant association with test modality was observed for OUES (*β* = 76, 95% CI −47 to 199, *p* = 0.224), VE/VCO₂ slope (*β* = 0.53, 95% CI −2.4 to 0.01, *p* = 0.382), or percent predicted oxygen pulse (*β* = 2.8, 95% CI −1.2 to 6.9, *p* = 0.173) (Tables 3, 4).

**Table 2:** This table summarizes demographic, clinical, CPET and CMR characteristics of the study population, stratified by sex (female and male) and exercise modality (bicycle vs. treadmill).

Different methods	F				M			
	Overall *n* = 128^a^	Bycicle *n* = 54^a^	Treadmill *n* = 74^a^	*P*-value^b^	Overall *n* = 162^a^	Bycicle *n* = 90^a^	Treadmill *n* = 72^a^	*P*-value^b^
Bmi (kg/m2)				0.377				0.690
Mean (SD)	22.4 (3.9)	22.8 (4.1)	22.2 (3.8)		23.3 (4.5)	23.6 (4.9)	23.0 (4.0)	
Median (Q1, Q3)	22.3 (19.8, 24.3)	22.7 (20.1, 24.8)	22.0 (19.8, 24.2)		23.1 (20.3, 25.8)	23.3 (19.9, 25.9)	22.3 (20.8, 25.6)	
IPAQ				0.855				0.830
0	22 (35%)	2 (29%)	20 (36%)		13 (23%)	1 (14%)	12 (24%)	
1	34 (55%)	4 (57%)	30 (55%)		39 (68%)	6 (86%)	33 (66%)	
2	6 (9.7%)	1 (14%)	5 (9.1%)		5 (8.8%)	0 (0%)	5 (10%)	
Age at CPET				0.264				0.045
Mean (SD)	23.5 (8.4)	24.5 (8.7)	22.8 (8.2)		22.7 (8.2)	23.7 (7.7)	21.6 (8.6)	
Median (Q1, Q3)	21.3 (16.5, 30.2)	21.1 (17.2, 32.8)	21.7 (15.6, 29.3)		21.6 (15.2, 28.7)	22.7 (17.5, 29.4)	19.0 (14.8, 26.5)	
VO2 peak (kg/mL/min)				**<0**.**001**				<**0**.**001**
Mean (SD)	25.6 (5.9)	23.4 (6.0)	27.2 (5.4)		28.9 (7.1)	26.5 (6.7)	31.8 (6.6)	
Median (Q1, Q3)	24.8 (21.9, 28.9)	22.3 (19.8, 25.1)	26.9 (23.7, 30.6)		28.4 (24.4, 33.0)	25.9 (22.0, 29.8)	31.8 (26.2, 36.0)	
VO2 pred (%)				**0**.**001**				**<0**.**001**
Mean (SD)	80.9 (15.1)	76.1 (13.6)	84.3 (15.3)		68.6 (14.3)	63.8 (13.5)	74.5 (13.0)	
Median (Q1, Q3)	80.1 (70.1, 91.0)	76.0 (64.6, 83.4)	82.2 (74.3, 93.2)		67.4 (58.6, 77.6)	63.2 (55.5, 71.0)	72.7 (64.6, 83.3)	
peak O₂ pulse (mL/beat)				**0**.**042**				0.076
Mean (SD)	8.7 (1.9)	8.3 (1.9)	9.0 (1.8)		11.5 (2.8)	11.1 (2.7)	11.9 (2.8)	
Median (Q1, Q3)	8.6 (7.3, 9.8)	8.0 (7.2, 9.3)	8.8 (7.5, 10.2)		11.4 (9.5, 13.2)	10.8 (9.4, 12.6)	11.9 (9.9, 13.8)	
peak O₂ pulse pred (%)				**0**.**047**				0.940
Mean (SD)	83.0 (13.9)	79.4 (11.4)	84.9 (14.8)		74.5 (15.3)	75.1 (16.9)	74.0 (13.7)	
Median (Q1, Q3)	81.8 (74.0, 93.0)	76.2 (71.6, 87.5)	84.1 (75.5, 95.6)		72.9 (64.0, 84.7)	73.0 (63.4, 85.8)	72.4 (66.0, 82.5)	
VE/VCO₂ slope at RCP				0.447				0.347
Mean (SD)	30.2 (4.6)	29.9 (4.8)	30.3 (4.4)		29.0 (4.8)	28.6 (4.7)	29.6 (4.8)	
Median (Q1, Q3)	29.2 (26.9, 33.0)	29.0 (26.5, 32.6)	29.6 (27.0, 33.4)		28.9 (25.4, 32.2)	28.3 (25.5, 31.5)	29.5 (25.2, 33.5)	
Oues (mL/min/min)				0.569				0.574
Mean (SD)	1,608.8 (375.8)	1,584.0 (397.5)	1,627.5 (360.5)		2,134.1 (582.7)	2,091.9 (545.7)	2,194.4 (631.5)	
Median (Q1, Q3)	1,582.0 (1,352.0, 1,820.0)	1,618.0 (1,261.0, 1,895.0)	1,578.5 (1,401.0, 1,815.0)		2,056.0 (1,678.0, 2,502.0)	2,061.5 (1,715.0, 2,388.0)	2,050.0 (1,664.0, 2,635.0)	
rvedvi (mL/m2)				0.231				0.534
Mean (SD)	115.4 (24.0)	113.1 (24.0)	117.1 (24.0)		123.1 (24.6)	124.1 (25.0)	121.8 (24.3)	
Median (Q1, Q3)	113.1 (100.6, 126.8)	111.9 (97.0, 125.2)	114.8 (104.0, 129.2)		122.1 (108.2, 137.7)	123.8 (110.3, 136.5)	119.3 (105.2, 139.8)	
rvesvi (mL/m2)				0.610				0.174
Mean (SD)	51.8 (15.4)	52.2 (14.8)	51.5 (15.9)		59.4 (15.5)	61.2 (16.8)	57.1 (13.6)	
Median (Q1, Q3)	51.1 (40.8, 59.6)	52.2 (41.6, 61.1)	51.1 (40.8, 58.2)		58.1 (48.1, 67.3)	58.5 (51.0, 67.8)	56.9 (46.6, 66.5)	
rvef (%)				**0**.**027**				**0**.**032**
Mean (SD)	55.4 (6.2)	54.0 (5.6)	56.4 (6.5)		52.3 (8.1)	51.5 (9.6)	53.3 (5.6)	
Median (Q1, Q3)	55.5 (50.4, 60.0)	53.9 (49.0, 58.0)	57.0 (51.0, 60.7)		51.4 (48.7, 55.8)	51.1 (47.1, 55.0)	53.5 (49.4, 56.3)	
lvef (%)				0.764				0.338
Mean (SD)	58.9 (6.5)	59.0 (5.3)	58.9 (7.3)		56.8 (7.6)	56.9 (9.3)	56.6 (4.6)	
Median (Q1, Q3)	58.9 (55.9, 62.7)	59.0 (55.9, 62.7)	58.6 (56.0, 62.7)		56.0 (53.4, 59.0)	56.0 (53.0, 58.5)	56.1 (54.0, 59.0)	
lvedvi (mL/m2)				0.109				**0.002**
Mean (SD)	78.8 (14.6)	80.7 (16.0)	77.3 (13.5)		85.6 (16.1)	89.1 (16.6)	81.2 (14.5)	
Median (Q1, Q3)	75.4 (69.9, 86.5)	79.4 (70.8, 89.3)	74.7 (68.8, 84.5)		85.8 (74.0, 95.1)	90.6 (78.0, 100.1)	79.9 (73.1, 89.9)	
lvesvi (mL/m2)				0.489				**0.004**
Mean (SD)	32.4 (7.9)	33.1 (8.6)	31.9 (7.3)		37.7 (9.5)	39.6 (10.1)	35.4 (8.3)	
Median (Q1, Q3)	31.2 (27.4, 36.5)	31.7 (27.5, 37.6)	30.9 (27.4, 36.2)		38.0 (31.4, 42.9)	39.7 (32.6, 46.3)	34.7 (29.1, 39.9)	
Pr (%)				**0**.**005**				**0**.**005**
Mean (SD)	24.6 (17.7)	19.3 (17.3)	28.5 (17.1)		21.6 (17.2)	18.2 (16.7)	25.9 (16.9)	
Median (Q1, Q3)	26.0 (5.9, 39.0)	15.7 (4.0, 34.0)	33.0 (12.9, 42.0)		19.3 (5.3, 36.0)	12.2 (5.2, 31.0)	29.5 (6.4, 40.5)	

IPAQ, International physical activity questionnaire; LVEDVI, left ventricular end-diastolic volume indexed to body surface area; LVESVI, left ventricular end-systolic volume indexed to body surface area; LVEF, Left ventricle ejection fraction; RVEF, Right ventricle ejection fraction; PR, Pulmonary regurgitation; RVEDVI, right ventricular end-diastolic volume indexed to body surface area; RVESVI, Right ventricular end-systolic volume indexed to body surface area; OUES, Oxygen uptake efficiency slope.

Statistically significant values (*p* < 0.05) are shown in bold.

^a^ *n* (%).

^b^ Wilcoxon rank sum test; Fisher's exact test.

**Table 3:** Multivariable regression results for absolute exercise parameters.

Variable	Parameter	*β* (coeff.)	95% CI	*P*-value
Modality (Treadmill vs. Bicycle)	VO₂ peak (mL/kg/min)	4.2	2.8–5.6	<0.001
	Oxygen pulse (mL/beat)	0.78	0.20–1.4	0.008
	VE/VCO₂ slope	0.53	−0.67 to 1.7	0.382
	OUES	76	−47 to 199	0.224
Sex (Male vs. Female)	VO₂ peak (mL/kg/min)	3.6	2.2–5.0	<0.001
	Oxygen pulse (mL/beat)	3.0	2.4–3.6	<0.001
	OUES	537	414–661	<0.001
	VE/VCO₂ slope	−1.2	−2.4 to 0.01	0.051
Age at CPET (years)	VO₂ peak (mL/kg/min)	−0.23	−0.32 to −0.15	<0.001
	Oxygen pulse (mL/beat)	0.04	0.01–0.08	0.016
	VE/VCO₂ slope	−0.08	−0.15 to 0.00	0.044
	OUES	−0.18	−7.5 to 7.1	0.962

**Table 4:** Regression results for percent predicted parameters.

Variable	Parameter	*β* (coeff.)	95% CI	*P*-value
Modality (treadmill vs. bicycle)	Percent predicted VO₂ peak	4.7	1.2–8.3	0.010
	Percent predicted oxygen pulse	2.8	−1.2 to 6.9	0.173

When the cohort was stratified by sex and age group (<18 and ≥18 years), no significant differences were observed between the two exercise modalities within the same sex and age category with respect to BMI, physical activity level, or NYHA class (Tables 5, 6). Notably, in the adult subgroup, both peak VO₂ and percent-predicted VO₂ values were significantly higher with treadmill testing in both males and females. Among younger patients, treadmill testing was associated with significantly higher peak VO₂ values in females, whereas no significant difference was found in males. Oxygen pulse also tended to be slightly higher in adult females during treadmill testing, while in males the difference did not reach statistical significance. VE/VCO₂ slope and OUES were not influenced by the type of exercise modality.

**Table 5:** Sex-specific comparison of cardiopulmonary, functional, and ventricular parameters in female patients with repaired Tetralogy of Fallot, stratified by age group (<18 years vs. ≥18 years) within each exercise modality (bicycle vs. treadmill).

Female	<18y				≥18y			
	Overall *n* = 42^a^	Bycicle *n* = 14^a^	Treadmill *n* = 28^a^	*P*-value^b^	Overall *n* = 86^a^	Bycicle *n* = 40^a^	Treadmill *n* = 46^a^	*P*-value^b^
bmi_kg_m2				0.463				0.609
Mean (SD)	21.2 (3.4)	21.5 (2.8)	21.0 (3.7)		23.0 (4.0)	23.2 (4.4)	22.9 (3.7)	
Median (Q1, Q3)	20.8 (18.9, 23.8)	21.1 (20.3, 23.2)	20.4 (17.9, 23.9)		22.8 (20.1, 24.7)	23.3 (19.9, 25.1)	22.7 (20.3, 24.2)	
IPAQ				0.329				0.720
0	9 (39%)	0 (0%)	9 (45%)		13 (33%)	2 (50%)	11 (31%)	
1	11 (48%)	2 (67%)	9 (45%)		23 (59%)	2 (50%)	21 (60%)	
2	3 (13%)	1 (33%)	2 (10%)		3 (7.7%)	0 (0%)	3 (8.6%)	
Age at CPET				0.420				0.753
Mean (SD)	14.8 (2.1)	15.2 (1.7)	14.5 (2.3)		27.8 (6.9)	27.8 (7.7)	27.8 (6.2)	
Median (Q1, Q3)	15.0 (13.6, 16.5)	15.6 (14.6, 16.4)	14.5 (12.6, 16.7)		26.4 (21.3, 34.2)	25.4 (20.9, 34.8)	26.7 (22.6, 34.0)	
VO2 peak (kg/mL/min)				**0**.**026**				<**0**.**001**
Mean (SD)	27.9 (5.9)	25.7 (6.4)	29.0 (5.4)		24.5 (5.7)	22.6 (5.8)	26.1 (5.1)	
Median (Q1, Q3)	27.6 (24.5, 32.0)	24.6 (21.3, 25.4)	29.0 (24.9, 32.5)		23.9 (21.4, 27.7)	21.6 (19.3, 24.7)	25.3 (23.1, 28.4)	
VO2 pred (%)				0.155				**0**.**003**
Mean (SD)	82.4 (16.1)	78.9 (14.9)	84.1 (16.7)		80.1 (14.6)	75.0 (13.2)	84.3 (14.5)	
Median (Q1, Q3)	80.4 (70.7, 91.9)	74.2 (68.6, 81.3)	81.9 (73.5, 95.2)		79.4 (68.3, 88.9)	76.7 (63.9, 83.5)	82.8 (77.4, 93.2)	
peak O₂ pulse (mL/beat)				0.540				**0**.**036**
Mean (SD)	8.5 (1.8)	8.1 (1.4)	8.6 (2.0)		8.8 (1.9)	8.4 (2.0)	9.1 (1.7)	
Median (Q1, Q3)	8.1 (7.3, 9.6)	8.0 (7.1, 9.3)	8.3 (7.3, 10.2)		8.8 (7.4, 9.9)	8.0 (7.2, 9.3)	9.1 (7.9, 10.2)	
peak O₂ pulse pred (%)				0.287				0.079
Mean (SD)	79.5 (13.7)	76.7 (8.5)	80.6 (15.3)		84.9 (13.7)	80.5 (12.4)	87.6 (14.0)	
Median (Q1, Q3)	76.8 (72.7, 92.2)	75.5 (72.5, 78.8)	78.4 (73.6, 95.0)		84.6 (74.5, 93.4)	80.5 (70.3, 89.5)	86.2 (77.5, 97.5)	
VE/VCO₂ slope at RCP				0.095				0.632
Mean (SD)	31.8 (4.3)	30.1 (3.2)	32.6 (4.6)		29.4 (4.5)	29.9 (5.2)	28.8 (3.5)	
Median (Q1, Q3)	31.5 (29.0, 34.5)	30.7 (28.0, 32.8)	33.1 (29.0, 35.5)		28.8 (26.4, 32.5)	28.9 (26.3, 32.6)	28.3 (26.4, 31.6)	
Oues (mL/min/min)				0.927				0.402
Mean (SD)	1,576.3 (335.0)	1,626.1 (364.8)	1,550.5 (323.1)		1,624.1 (394.5)	1,569.6 (411.7)	1,672.3 (376.9)	
Median (Q1, Q3)	1,567.5 (1,354.0, 1,792.0)	1,632.0 (1,337.0, 1,718.0)	1,554.0 (1,380.0, 1,820.0)		1,618.0 (1,352.0, 1,820.0)	1,600.0 (1,230.0, 1,923.0)	1,631.0 (1,411.0, 1,810.0)	
rvedvi (mL/m2)				0.843				0.271
Mean (SD)	116.3 (22.6)	112.9 (17.9)	117.9 (24.8)		115.0 (24.8)	113.2 (26.0)	116.5 (23.8)	
Median (Q1, Q3)	114.5 (104.0, 126.7)	114.0 (97.0, 125.2)	115.4 (104.7, 129.5)		112.1 (100.2, 126.8)	111.2 (96.6, 124.2)	114.8 (102.6, 129.2)	
rvesvi (mL/m2)				0.189				0.858
Mean (SD)	51.8 (14.1)	54.0 (10.0)	50.7 (15.8)		51.8 (16.1)	51.5 (16.2)	52.0 (16.1)	
Median (Q1, Q3)	49.4 (42.4, 58.8)	55.0 (48.3, 62.4)	47.5 (40.6, 56.3)		51.9 (40.7, 59.6)	51.0 (40.3, 60.5)	52.0 (41.3, 58.3)	
rvef (%)				**0**.**003**				0.495
Mean (SD)	55.7 (6.0)	52.3 (3.9)	57.5 (6.1)		55.2 (6.4)	54.6 (6.0)	55.7 (6.8)	
Median (Q1, Q3)	56.4 (52.0, 60.0)	52.8 (48.5, 54.1)	57.7 (54.9, 61.2)		55.0 (50.4, 60.0)	54.5 (50.2, 58.7)	55.1 (50.4, 60.0)	
lvef (%)				0.936				0.517
Mean (SD)	60.0 (8.3)	59.0 (5.7)	60.5 (9.3)		58.4 (5.5)	59.0 (5.2)	57.9 (5.7)	
Median (Q1, Q3)	60.3 (56.0, 63.0)	60.7 (55.8, 63.3)	59.0 (56.0, 63.0)		58.0 (55.3, 62.0)	58.6 (56.0, 62.4)	57.5 (55.0, 61.0)	
lvedvi (mL/m2)				0.607				0.055
Mean (SD)	78.0 (10.7)	76.7 (7.7)	78.7 (12.0)		79.1 (16.2)	82.2 (17.9)	76.5 (14.3)	
Median (Q1, Q3)	75.1 (70.6, 86.6)	73.8 (71.6, 81.5)	75.6 (70.5, 87.7)		75.6 (69.0, 86.1)	81.0 (70.6, 95.5)	71.3 (68.2, 83.3)	
lvesvi (mL/m2				0.321				0.142
Mean (SD)	31.8 (6.8)	30.2 (5.6)	32.6 (7.2)		32.7 (8.4)	34.2 (9.2)	31.5 (7.4)	
Median (Q1, Q3)	31.0 (27.5, 35.8)	30.0 (25.3, 33.4)	31.7 (28.0, 36.7)		31.3 (27.4, 36.6)	31.8 (27.8, 39.3)	30.7 (27.2, 35.5)	
Pr (%)				0.109				**0.044**
Mean (SD)	28.2 (18.0)	22.0 (19.6)	31.2 (16.8)		22.9 (17.4)	18.4 (16.7)	26.8 (17.2)	
Median (Q1, Q3)	31.6 (9.4, 41.5)	22.4 (4.0, 35.0)	34.0 (23.0, 44.0)		20.0 (5.7, 38.8)	14.2 (4.0, 28.3)	33.0 (10.3, 39.0)	

IPAQ, International physical activity questionnaire; LVEDVI, left ventricular end-diastolic volume indexed to body surface area; LVESVI, left ventricular end-systolic volume indexed to body surface area; LVEF, Left ventricle ejection fraction; RVEF, Right ventricle ejection fraction; PR, Pulmonary regurgitation; RVEDVI, right ventricular end-diastolic volume indexed to body surface area; RVESVI, Right ventricular end-systolic volume indexed to body surface area; OUES, Oxygen uptake efficiency slope.

^a^ *n* (%).

^b^ Wilcoxon rank sum test; Fisher's exact test; Wilcoxon rank sum exact test.

**Table 6:** Sex-specific comparison of cardiopulmonary, functional, and ventricular parameters in male patients with repaired Tetralogy of Fallot, stratified by age group (<18 years vs. ≥18 years) within each exercise modality (bicycle vs. treadmill).

Male	<18y				≥18y			
	Overall *n* = 61^a^	Bycicle *n* = 27^a^	Treadmill *n* = 34^a^	*P*-value^b^	Overall *n* = 100^a^	Bycicle *n* = 62^a^	Treadmill *n* = 38^a^	*P*-value^c^
Bmi (kg/m2)				0.880				0.877
Mean (SD)	22.2 (4.1)	22.3 (4.3)	22.2 (3.9)		24.0 (4.7)	24.2 (5.1)	23.8 (3.9)	
Median (Q1, Q3)	21.9 (19.0, 24.8)	21.9 (18.4, 25.9)	21.9 (19.3, 24.5)		23.3 (21.5, 26.1)	23.5 (21.3, 25.9)	23.2 (21.9, 26.3)	
IPAQ				0.539				>0.999
0	6 (26%)	0 (0%)	6 (30%)		7 (21%)	1 (25%)	6 (20%)	
1	17 (74%)	3 (100%)	14 (70%)		22 (65%)	3 (75%)	19 (63%)	
2	0 (0%)	0 (0%)	0 (0%)		5 (15%)	0 (0%)	5 (17%)	
Age at CPET				0.242				0.817
Mean (SD)	14.7 (1.8)	15.0 (1.8)	14.4 (1.8)		27.7 (6.4)	27.5 (6.1)	28.0 (7.0)	
Median (Q1, Q3)	14.6 (13.4, 15.8)	14.6 (13.6, 16.9)	14.7 (13.1, 15.4)		26.1 (22.5, 32.5)	26.2 (22.3, 31.7)	25.7 (23.0, 32.6)	
VO2 peak (kg/mL/min)				0.062				**<0**.**001**
Mean (SD)	32.1 (7.7)	30.4 (8.2)	33.5 (7.1)		26.9 (6.0)	24.8 (5.2)	30.3 (5.8)	
Median (Q1, Q3)	31.5 (26.1, 36.6)	28.6 (24.5, 33.3)	33.7 (29.1, 37.4)		26.2 (22.8, 31.3)	25.2 (21.2, 28.0)	31.3 (25.2, 33.6)	
VO2 pred (%)				0.085				**<0**.**001**
Mean (SD)	72.0 (14.4)	68.3 (13.6)	75.0 (14.5)		66.5 (13.9)	61.8 (13.1)	74.1 (11.6)	
Median (Q1, Q3)	70.0 (62.1, 80.5)	67.7 (59.8, 77.1)	72.5 (63.4, 84.3)		65.9 (56.7, 74.4)	59.3 (53.6, 69.5)	72.7 (66.0, 82.3)	
peak O₂ pulse (mL/beat)				0.303				0.095
Mean (SD)	11.0 (2.7)	10.5 (2.0)	11.5 (3.2)		11.8 (2.8)	11.5 (2.9)	12.4 (2.5)	
Median (Q1, Q3)	10.5 (9.2, 12.7)	10.1 (9.2, 11.9)	10.8 (9.1, 13.8)		11.8 (10.0, 13.4)	11.5 (9.9, 13.2)	12.2 (10.7, 14.1)	
peak O₂ pulse pred (%)				0.405				0.421
Mean (SD)	73.4 (15.3)	70.7 (15.8)	74.9 (15.1)		75.4 (15.3)	77.3 (17.3)	73.2 (12.5)	
Median (Q1, Q3)	73.0 (65.5, 81.6)	70.9 (59.0, 76.9)	73.0 (65.6, 82.5)		72.9 (64.1, 85.8)	76.8 (63.8, 90.2)	72.1 (66.2, 84.2)	
VE/VCO₂ slope at RCP				0.761				0.688
Mean (SD)	30.3 (4.7)	30.0 (4.0)	30.7 (5.3)		28.1 (4.7)	28.0 (4.9)	28.4 (4.1)	
Median (Q1, Q3)	30.0 (26.8, 33.7)	30.0 (26.9, 32.6)	30.5 (26.5, 33.8)		27.8 (25.0, 30.9)	27.2 (25.0, 30.7)	28.6 (25.0, 32.0)	
Oues (mL/min/min)				0.436				0.184
Mean (SD)	2,182.1 (595.8)	2,189.3 (487.9)	2,175.8 (684.1)		2,104.8 (578.9)	2,049.2 (571.8)	2,212.4 (586.5)	
Median (Q1, Q3)	2,096.0 (1,674.0, 2,647.0)	2,180.0 (1,812.0, 2,647.0)	1,964.0 (1,657.0, 2,694.0)		2,006.5 (1,692.0, 2,441.0)	1,945.5 (1,677.0, 2,275.0)	2,104.0 (1,738.0, 2,582.5)	
rvedvi (mL/m2)				0.970				0.346
Mean (SD)	128.5 (23.9)	129.3 (18.1)	127.8 (27.9)		119.2 (24.2)	121.0 (26.7)	116.1 (19.1)	
Median (Q1, Q3)	127.6 (113.6, 141.5)	124.3 (117.4, 142.3)	129.3 (106.8, 141.4)		118.3 (105.4, 134.1)	122.8 (107.1, 135.9)	113.5 (105.0, 127.1)	
rvesvi (mL/m2)				0.426				0.322
Mean (SD)	59.4 (13.7)	61.2 (11.7)	58.0 (15.2)		59.2 (16.6)	61.0 (18.7)	56.3 (12.1)	
Median (Q1, Q3)	59.0 (50.7, 67.1)	59.4 (54.4, 67.8)	59.0 (46.2, 66.5)		56.6 (47.8, 67.3)	57.6 (48.1, 67.4)	54.4 (46.8, 66.5)	
rvef (%)				0.076				0.324
Mean (SD)	53.6 (5.1)	52.3 (5.0)	54.7 (4.9)		51.5 (9.5)	51.1 (11.2)	52.0 (5.8)	
Median (Q1, Q3)	53.7 (50.1, 57.8)	51.4 (50.0, 55.0)	54.3 (51.0, 58.3)		51.0 (47.0, 55.0)	50.8 (46.0, 55.0)	51.0 (48.0, 55.7)	
lvef (%)				0.057				0.491
Mean (SD)	57.7 (4.7)	56.5 (5.0)	58.6 (4.2)		56.1 (8.9)	57.0 (10.7)	54.7 (4.2)	
Median (Q1, Q3)	57.0 (54.7, 61.0)	56.0 (53.7, 57.8)	58.0 (55.0, 62.3)		55.4 (53.0, 58.0)	55.6 (52.7, 58.5)	55.0 (53.0, 57.0)	
lvedvi (mL/m2)				0.104				**0**.**015**
Mean (SD)	82.5 (14.9)	86.2 (15.2)	79.6 (14.1)		87.5 (16.7)	90.5 (17.2)	82.6 (14.9)	
Median (Q1, Q3)	80.6 (72.7, 93.0)	88.0 (72.8, 95.3)	79.7 (72.5, 89.0)		88.8 (75.7, 97.4)	92.2 (80.3, 101.2)	81.0 (73.3, 91.2)	
lvesvi (mL/m2)				**0.010**				0.160
Mean (SD)	35.5 (8.4)	38.6 (8.1)	33.0 (7.8)		39.2 (10.0)	40.1 (10.9)	37.7 (8.3)	
Median (Q1, Q3)	35.7 (28.8, 40.6)	38.5 (33.3, 42.8)	31.4 (27.7, 38.2)		39.3 (32.4, 44.9)	40.1 (31.9, 46.9)	37.4 (32.7, 41.2)	
Pr (%)				0.278				**0**.**029**
Mean (SD)	26.3 (16.7)	23.7 (16.4)	28.3 (17.0)		18.5 (16.9)	15.3 (16.3)	23.6 (16.8)	
Median (Q1, Q3)	27.0 (9.0, 43.0)	22.5 (7.0, 35.5)	33.0 (12.3, 43.0)		12.2 (4.8, 31.6)	8.8 (4.5, 21.9)	29.0 (4.8, 38.0)	

IPAQ, International physical activity questionnaire; LVEDVI, left ventricular end-diastolic volume indexed to body surface area; LVESVI, left ventricular end-systolic volume indexed to body surface area; LVEF, Left ventricle ejection fraction; RVEF, Right ventricle ejection fraction; PR, Pulmonary regurgitation; RVEDVI, right ventricular end-diastolic volume indexed to body surface area; RVESVI, Right ventricular end-systolic volume indexed to body surface area; OUES, Oxygen uptake efficiency slope.

^a^ *n* (%).

^b^ Wilcoxon rank sum exact test; Fisher's exact test; Wilcoxon rank sum test.

^c^ Wilcoxon rank sum test; Fisher's exact test; Wilcoxon rank sum exact test.

When analyzing age-related differences within the same exercise modality, we found that, unlike in females—where both peak VO₂ and its percent-predicted value were significantly lower in older patients across both modalities (Supplementary Table S1)—in males, this difference was observed only in the cycle ergometer group (Supplementary Table S2). Notably, although a trend toward lower peak VO₂ values was observed in older males undergoing treadmill testing, the difference did not reach statistical significance. Interestingly, oxygen pulse, VE/VCO₂ slope, and OUES did not differ significantly between age groups in either modality.

## 4 Discussion

Our study, conducted in asymptomatic rToF patients—most of whom were physically active—confirms the safety and feasibility of both treadmill and cycle ergometer testing for evaluating functional capacity. By analysing a larger cohort, we confirmed our previous findings (11) that the choice of exercise modality significantly influences CPET results and must be clearly reported to ensure reliable longitudinal evaluation. In both sexes, among patients with NYHA class I and preserved ventricular function, treadmill testing consistently yielded higher VO₂ peak values than cycle ergometry. This finding is consistent with our previous study (11), in which the two modalities were compared within the same individuals, and is also in line with prior evidence in both healthy individuals and patients with cardiovascular disease (7, 8). The choice between treadmill and cycle ergometer has a significant impact on test results, as the difference in oxygen consumption between the two modalities can range from 10% to 20%, with higher values typically observed during treadmill testing. This must be considered when interpreting the comprehensive clinical and instrumental assessment of each patient. This issue is particularly relevant in rToF population, where a percent-predicted VO₂ of 70% is frequently used as one of the thresholds to consider pulmonary valve replacement (PVR). Several studies have shown that a predicted VO₂ around 70% is commonly found in well-compensated adults with rToF and preserved ejection fraction (4, 6, 14, 15). Consequently, not only should the appropriateness of using this threshold as a decision-making criterion for PVR be critically reassessed, but it is also essential to clearly specify the exercise modality from which the VO₂ measurement was obtained. Indeed, although VO₂ peak is the most used parameter in exercise testing to assess a patient's functional capacity, it is also among the most complex to interpret accurately. It is influenced by multiple factors—including age, BMI, sex (16), pulmonary function, muscle mass, exercise modality, haemoglobin levels, and the patient's level of physical conditioning. In addition, oxygen consumption is also influenced by the patient's level of cooperation and their familiarity with the exercise modality being used. Finally, in patients younger than 18 years, we should also consider puberty that introduces sex-specific changes at varying ages, including increased muscle mass, higher oxygen-carrying capacity and larger heart size in males (17). Therefore, its interpretation requires careful consideration of all variables that may affect the result, to obtain a value that truly reflects the patient's functional status. This is particularly important, as in this population—as in other cardiac conditions—VO₂ peak, along with VE/VCO₂ slope and heart rate reserve, are established predictors of adverse outcomes, including early mortality (18–21). Regarding oxygen pulse, which has been poorly described in rToF patients, we believe that this parameter may provide valuable insight into the patient's true cardiovascular function, as it is calculated as the ratio between oxygen consumption and heart rate—and an abnormal chronotropic response to exercise with advancing age is well recognized in this population. The apparent discrepancy between the univariate and multivariate analyses observed in our study may be explained by the interplay between exercise modality, sex, and age. In univariate analysis, significant differences in both absolute and percent-predicted oxygen pulse were observed only in females, likely reflecting sex-related differences in heart rate dynamics and exercise efficiency (16, 22). Women tend to achieve higher heart rates and exhibit a slower age-related decline in chronotropic reserve, which may accentuate the treadmill effect on oxygen pulse. When sex and age were included as covariates in the multivariate model, the absolute peak oxygen pulse remained significantly influenced by the exercise modality, whereas the percent-predicted value did not. This finding suggests that the observed difference is primarily driven by physiological variations in heart rate response rather than by intrinsic differences in exercise performance once normalized for sex and age. Unlike oxygen pulse, OUES was not significantly affected by exercise modality after adjustment for age and sex. This finding is physiologically consistent, since OUES—being a submaximal parameter reflecting the efficiency of oxygen uptake relative to ventilation—is less dependent on maximal heart rate response or cardiac output. Consequently, it tends to remain stable across different exercise modalities once demographic variables are accounted for, indicating that OUES primarily reflects the integrated ventilatory–metabolic response rather than the cardiovascular performance *per se*. This finding warrants further investigation in larger and more precisely characterized cohorts, ideally including an assessment of daily physical activity. OUES may represent a particularly valuable parameter in this population (23), as it does not require maximal effort and is therefore easier to obtain even in deconditioned or symptomatic patients. By integrating both cardiopulmonary reserve and ventilatory efficiency, OUES could provide a more comprehensive and realistic assessment of functional capacity in individuals with rToF. Finally, the VE/VCO₂ slope did not differ significantly between treadmill and cycle ergometer testing, confirming the findings of our previous study (11). Notably, the Bruce treadmill protocol—despite involving less consistent, larger, and irregular workload increments—can still yield an accurate assessment of the VE/VCO₂ slope, comparable to that obtained with cycle ergometry. This finding supports the use of treadmill testing, which more closely resembles the patient's daily physical activity. In contrast, cycle ergometer testing may be less representative of overall functional capacity, particularly in patients who are not specifically trained for lower-limb-dominant exercise. Moreover, in rToF patients—where chronotropic incompetence may occur in response to increasing workloads during exercise testing—treadmill protocols may be more appropriate for detecting subtle changes in functional capacity, as they tend to elicit a greater increase in heart rate compared to cycle ergometry. Interestingly, we did not observe higher VE/VCO₂ slope values in older patients when considering the same exercise modality, in either males or females, despite well-established evidence in the literature indicating that the ventilatory response to increasing CO₂ production during exercise (as reflected by the VE/VCO₂ slope) tends to progressively increase after the second decade of life (24). This last result could be due to differences in the composition of the study populations, as this parameter is highly influenced by the heterogeneity of age, the presence of underlying pulmonary conditions, and ventricular function.

### 4.1 Limitations

The present study included a relatively young patient population, with only a limited number of participants older than 40 years. In addition, right ventricular systolic function was largely preserved across the cohort, as no patient had a right ventricular ejection fraction (RVEF) below 45%. Therefore, the results may not be fully generalizable to older patients or to individuals with more advanced right ventricular dysfunction. Moreover, most patients reported engaging in at least some degree of habitual physical activity. As a consequence, the findings may not accurately reflect cardiopulmonary responses in sedentary individuals with Tetralogy of Fallot. Physical activity level was assessed using the IPAQ questionnaire and did not differ significantly between exercise modality groups. Multivariable analyses were performed to evaluate the independent association between CPET parameters and exercise modality after adjustment for sex, age, body surface area, and IPAQ score. However, a non-random allocation of exercise modality cannot be completely excluded. This limitation is inherent to the between-subject study design, as treadmill and cycle ergometer tests were not performed within the same individuals. Accordingly, a potential selection bias should be considered, even though treadmill testing is generally preferred at our institution. Patients with higher physical fitness may have been more likely to undergo treadmill testing, whereas differences in body habitus, age, or lower-limb strength may have influenced referral to cycle ergometer testing.

## 5 Conclusions

We stratified the cardiopulmonary exercise testing (CPET) parameters currently considered most relevant in Tetralogy of Fallot by sex, age, and the exercise modality used to perform the test. Our study aims to provide an overview of the values that can be expected in a well-compensated rToF population—most of whom are at least minimally physically active—within specific age and sex groups, and according to the testing modality. All these data points (home physical activity, MRI parameters, NYHA class, and exercise modality) contribute to a more comprehensive evaluation of the patient's overall condition.

## Data Availability

The datasets presented in this article are not readily available because our hospital policy does not allow the sharing of any patient data. Requests to access the datasets should be directed to https://www.ospedalebambinogesu.it.
