# Supplementary material for: Cardiopulmonary parameters in patients with Tetralogy of Fallot: the reference values for treadmill and cycle ergometer
Source: Front Cardiovasc Med. 2026 Apr 21;13:1673478. doi: 10.3389/fcvm.2026.1673478 (PMC13139358; doi:10.3389/fcvm.2026.1673478)
Supplement: Supplementary file 1 [file Table1.docx]

|  | Bycicle | | | | Treadmill | | | |
| --- | --- | --- | --- | --- | --- | --- | --- | --- |
| **Female** | **Overall**  N = 54^1^ | **<18y**  N = 14^1^ | **≥18y**  N = 40^1^ | **p-value**^2^ | **Overall**  N = 74^1^ | **<18y**  N = 28^1^ | **≥18y**  N = 46^1^ | **p-value**^2^ |
| Bmi (kg/m2) |  |  |  | 0.189 |  |  |  | 0.032 |
| Mean (SD) | 22.8 (4.1) | 21.5 (2.8) | 23.2 (4.4) |  | 22.2 (3.8) | 21.0 (3.7) | 22.9 (3.7) |  |
| Median (Q1, Q3) | 22.7 (20.1, 24.8) | 21.1 (20.3, 23.2) | 23.3 (19.9, 25.1) |  | 22.0 (19.8, 24.2) | 20.4 (17.9, 23.9) | 22.7 (20.3, 24.2) |  |
| IPAQ |  |  |  | 0.429 |  |  |  | 0.590 |
| 0 | 2 (29%) | 0 (0%) | 2 (50%) |  | 20 (36%) | 9 (45%) | 11 (31%) |  |
| 1 | 4 (57%) | 2 (67%) | 2 (50%) |  | 30 (55%) | 9 (45%) | 21 (60%) |  |
| 2 | 1 (14%) | 1 (33%) | 0 (0%) |  | 5 (9.1%) | 2 (10%) | 3 (8.6%) |  |
| Age at CPET |  |  |  | **<0.001** |  |  |  | **<0.001** |
| Mean (SD) | 24.5 (8.7) | 15.2 (1.7) | 27.8 (7.7) |  | 22.8 (8.2) | 14.5 (2.3) | 27.8 (6.2) |  |
| Median (Q1, Q3) | 21.1 (17.2, 32.8) | 15.6 (14.6, 16.4) | 25.4 (20.9, 34.8) |  | 21.7 (15.6, 29.3) | 14.5 (12.6, 16.7) | 26.7 (22.6, 34.0) |  |
| VO2 peak (kg/ml/min) |  |  |  | 0.056 |  |  |  | **0.005** |
| Mean (SD) | 23.4 (6.0) | 25.7 (6.4) | 22.6 (5.8) |  | 27.2 (5.4) | 29.0 (5.4) | 26.1 (5.1) |  |
| Median (Q1, Q3) | 22.3 (19.8, 25.1) | 24.6 (21.3, 25.4) | 21.6 (19.3, 24.7) |  | 26.9 (23.7, 30.6) | 29.0 (24.9, 32.5) | 25.3 (23.1, 28.4) |  |
| VO2 pred (%) |  |  |  | 0.547 |  |  |  | 0.925 |
| Mean (SD) | 76.1 (13.6) | 78.9 (14.9) | 75.0 (13.2) |  | 84.3 (15.3) | 84.1 (16.7) | 84.3 (14.5) |  |
| Median (Q1, Q3) | 76.0 (64.6, 83.4) | 74.2 (68.6, 81.3) | 76.7 (63.9, 83.5) |  | 82.2 (74.3, 93.2) | 81.9 (73.5, 95.2) | 82.8 (77.4, 93.2) |  |
| peak O₂ pulse (mL/beat) |  |  |  | 0.803 |  |  |  | 0.230 |
| Mean (SD) | 8.3 (1.9) | 8.1 (1.4) | 8.4 (2.0) |  | 9.0 (1.8) | 8.6 (2.0) | 9.1 (1.7) |  |
| Median (Q1, Q3) | 8.0 (7.2, 9.3) | 8.0 (7.1, 9.3) | 8.0 (7.2, 9.3) |  | 8.8 (7.5, 10.2) | 8.3 (7.3, 10.2) | 9.1 (7.9, 10.2) |  |
| peak O₂ pulse pred (%) |  |  |  | 0.401 |  |  |  | 0.078 |
| Mean (SD) | 79.4 (11.4) | 76.7 (8.5) | 80.5 (12.4) |  | 84.9 (14.8) | 80.6 (15.3) | 87.6 (14.0) |  |
| Median (Q1, Q3) | 76.2 (71.6, 87.5) | 75.5 (72.5, 78.8) | 80.5 (70.3, 89.5) |  | 84.1 (75.5, 95.6) | 78.4 (73.6, 95.0) | 86.2 (77.5, 97.5) |  |
| VE/VCO₂ slope at RCP |  |  |  | 0.442 |  |  |  | **0.002** |
| Mean (SD) | 29.9 (4.8) | 30.1 (3.2) | 29.9 (5.2) |  | 30.3 (4.4) | 32.6 (4.6) | 28.8 (3.5) |  |
| Median (Q1, Q3) | 29.0 (26.5, 32.6) | 30.7 (28.0, 32.8) | 28.9 (26.3, 32.6) |  | 29.6 (27.0, 33.4) | 33.1 (29.0, 35.5) | 28.3 (26.4, 31.6) |  |
| Oues (ml/min/min) |  |  |  | 0.854 |  |  |  | 0.366 |
| Mean (SD) | 1,584.0 (397.5) | 1,626.1 (364.8) | 1,569.6 (411.7) |  | 1,627.5 (360.5) | 1,550.5 (323.1) | 1,672.3 (376.9) |  |
| Median (Q1, Q3) | 1,618.0 (1,261.0, 1,895.0) | 1,632.0 (1,337.0, 1,718.0) | 1,600.0 (1,230.0, 1,923.0) |  | 1,578.5 (1,401.0, 1,815.0) | 1,554.0 (1,380.0, 1,820.0) | 1,631.0 (1,411.0, 1,810.0) |  |
| rvedvi (ml/m2) |  |  |  | 0.661 |  |  |  | 0.897 |
| Mean (SD) | 113.1 (24.0) | 112.9 (17.9) | 113.2 (26.0) |  | 117.1 (24.0) | 117.9 (24.8) | 116.5 (23.8) |  |
| Median (Q1, Q3) | 111.9 (97.0, 125.2) | 114.0 (97.0, 125.2) | 111.2 (96.6, 124.2) |  | 114.8 (104.0, 129.2) | 115.4 (104.7, 129.5) | 114.8 (102.6, 129.2) |  |
| rvesvi (ml/m2) |  |  |  | 0.373 |  |  |  | 0.369 |
| Mean (SD) | 52.2 (14.8) | 54.0 (10.0) | 51.5 (16.2) |  | 51.5 (15.9) | 50.7 (15.8) | 52.0 (16.1) |  |
| Median (Q1, Q3) | 52.2 (41.6, 61.1) | 55.0 (48.3, 62.4) | 51.0 (40.3, 60.5) |  | 51.1 (40.8, 58.2) | 47.5 (40.6, 56.3) | 52.0 (41.3, 58.3) |  |
| rvef (%) |  |  |  | 0.144 |  |  |  | 0.204 |
| Mean (SD) | 54.0 (5.6) | 52.3 (3.9) | 54.6 (6.0) |  | 56.4 (6.5) | 57.5 (6.1) | 55.7 (6.8) |  |
| Median (Q1, Q3) | 53.9 (49.0, 58.0) | 52.8 (48.5, 54.1) | 54.5 (50.2, 58.7) |  | 57.0 (51.0, 60.7) | 57.7 (54.9, 61.2) | 55.1 (50.4, 60.0) |  |
| lvef (%) |  |  |  | 0.635 |  |  |  | 0.242 |
| Mean (SD) | 59.0 (5.3) | 59.0 (5.7) | 59.0 (5.2) |  | 58.9 (7.3) | 60.5 (9.3) | 57.9 (5.7) |  |
| Median (Q1, Q3) | 59.0 (55.9, 62.7) | 60.7 (55.8, 63.3) | 58.6 (56.0, 62.4) |  | 58.6 (56.0, 62.7) | 59.0 (56.0, 63.0) | 57.5 (55.0, 61.0) |  |
| lvedvi (ml/m2) |  |  |  | 0.266 |  |  |  | 0.152 |
| Mean (SD) | 80.7 (16.0) | 76.7 (7.7) | 82.2 (17.9) |  | 77.3 (13.5) | 78.7 (12.0) | 76.5 (14.3) |  |
| Median (Q1, Q3) | 79.4 (70.8, 89.3) | 73.8 (71.6, 81.5) | 81.0 (70.6, 95.5) |  | 74.7 (68.8, 84.5) | 75.6 (70.5, 87.7) | 71.3 (68.2, 83.3) |  |
| lvesvi (ml/m2) |  |  |  | 0.182 |  |  |  | 0.358 |
| Mean (SD) | 33.1 (8.6) | 30.2 (5.6) | 34.2 (9.2) |  | 31.9 (7.3) | 32.6 (7.2) | 31.5 (7.4) |  |
| Median (Q1, Q3) | 31.7 (27.5, 37.6) | 30.0 (25.3, 33.4) | 31.8 (27.8, 39.3) |  | 30.9 (27.4, 36.2) | 31.7 (28.0, 36.7) | 30.7 (27.2, 35.5) |  |
| Pr (%) |  |  |  | 0.828 |  |  |  | 0.323 |
| Mean (SD) | 19.3 (17.3) | 22.0 (19.6) | 18.4 (16.7) |  | 28.5 (17.1) | 31.2 (16.8) | 26.8 (17.2) |  |
| Median (Q1, Q3) | 15.7 (4.0, 34.0) | 22.4 (4.0, 35.0) | 14.2 (4.0, 28.3) |  | 33.0 (12.9, 42.0) | 34.0 (23.0, 44.0) | 33.0 (10.3, 39.0) |  |
| ^1^n (%) | | | | | | | | |
| ^2^Wilcoxon rank sum test; Fisher's exact test; Wilcoxon rank sum exact test | | | | | | | | |

*Supplementary Table 1*: Baseline anthropometric, functional, and ventricular parameters in female patients stratified by exercise modality (bicycle vs treadmill) and age group (<18 years and ≥18 years). Legend: BMI = body mass index; IPAQ = International Physical Activity Questionnaire; CPET = cardiopulmonary exercise test; LVEDVI = left ventricular end-diastolic volume indexed to body surface area (BSA); LVESVI = left ventricular end-systolic volume indexed to BSA; LVEF = left ventricular ejection fraction; OUES = oxygen uptake efficiency slope RVEDVI = right ventricular end-diastolic volume indexed to BSA; RVESVI = right ventricular end-systolic volume indexed to BSA; RVEF = right ventricular ejection fraction; PR = pulmonary regurgitation; VO₂ = oxygen uptake.
