# Supplementary material for: Cardiopulmonary parameters in patients with Tetralogy of Fallot: the reference values for treadmill and cycle ergometer
Source: Front Cardiovasc Med. 2026 Apr 21;13:1673478. doi: 10.3389/fcvm.2026.1673478 (PMC13139358; doi:10.3389/fcvm.2026.1673478)
Supplement: Supplementary file 2 [file Table2.docx]

|  | Bycicle | | | | Treadmill | | | |
| --- | --- | --- | --- | --- | --- | --- | --- | --- |
| **Male** | **Overall**  N = 89^1^ | **<18y**  N = 27^1^ | **≥18y**  N = 62^1^ | **p-value**^2^ | **Overall**  N = 72^1^ | **<18y**  N = 34^1^ | **≥18y**  N = 38^1^ | **p-value**^2^ |
| bmi_kg_m2 |  |  |  | 0.105 |  |  |  | 0.120 |
| Mean (SD) | 23.6 (4.9) | 22.3 (4.3) | 24.2 (5.1) |  | 23.0 (4.0) | 22.2 (3.9) | 23.8 (3.9) |  |
| Median (Q1, Q3) | 23.3 (19.9, 25.9) | 21.9 (18.4, 25.9) | 23.5 (21.3, 25.9) |  | 22.3 (20.8, 25.6) | 21.9 (19.3, 24.5) | 23.2 (21.9, 26.3) |  |
| IPAQ |  |  |  | >0.999 |  |  |  | 0.166 |
| 0 | 1 (14%) | 0 (0%) | 1 (25%) |  | 12 (24%) | 6 (30%) | 6 (20%) |  |
| 1 | 6 (86%) | 3 (100%) | 3 (75%) |  | 33 (66%) | 14 (70%) | 19 (63%) |  |
| 2 | 0 (0%) | 0 (0%) | 0 (0%) |  | 5 (10%) | 0 (0%) | 5 (17%) |  |
| Age at CPET |  |  |  | **<0.001** |  |  |  | **<0.001** |
| Mean (SD) | 23.7 (7.7) | 15.0 (1.8) | 27.5 (6.1) |  | 21.6 (8.6) | 14.4 (1.8) | 28.0 (7.0) |  |
| Median (Q1, Q3) | 22.7 (17.5, 29.4) | 14.6 (13.6, 16.9) | 26.2 (22.3, 31.7) |  | 19.0 (14.8, 26.5) | 14.7 (13.1, 15.4) | 25.7 (23.0, 32.6) |  |
| VO2 peak (kg/ml/min) |  |  |  | **0.003** |  |  |  | 0.063 |
| Mean (SD) | 26.5 (6.7) | 30.4 (8.2) | 24.8 (5.2) |  | 31.8 (6.6) | 33.5 (7.1) | 30.3 (5.8) |  |
| Median (Q1, Q3) | 25.8 (22.0, 29.8) | 28.6 (24.5, 33.3) | 25.2 (21.2, 28.0) |  | 31.8 (26.2, 36.0) | 33.7 (29.1, 37.4) | 31.3 (25.2, 33.6) |  |
| VO2 pred (%) |  |  |  | 0.020 |  |  |  | 0.969 |
| Mean (SD) | 63.8 (13.5) | 68.3 (13.6) | 61.8 (13.1) |  | 74.5 (13.0) | 75.0 (14.5) | 74.1 (11.6) |  |
| Median (Q1, Q3) | 63.2 (55.5, 71.0) | 67.7 (59.8, 77.1) | 59.3 (53.6, 69.5) |  | 72.7 (64.6, 83.3) | 72.5 (63.4, 84.3) | 72.7 (66.0, 82.3) |  |
| peak O₂ pulse (mL/beat) |  |  |  | 0.125 |  |  |  | 0.095 |
| Mean (SD) | 11.2 (2.7) | 10.5 (2.0) | 11.5 (2.9) |  | 11.9 (2.8) | 11.5 (3.2) | 12.4 (2.5) |  |
| Median (Q1, Q3) | 10.8 (9.4, 12.7) | 10.1 (9.2, 11.9) | 11.5 (9.9, 13.2) |  | 11.9 (9.9, 13.8) | 10.8 (9.1, 13.8) | 12.2 (10.7, 14.1) |  |
| peak O₂ pulse pred (%) |  |  |  | 0.201 |  |  |  | 0.819 |
| Mean (SD) | 75.3 (17.0) | 70.7 (15.8) | 77.3 (17.3) |  | 74.0 (13.7) | 74.9 (15.1) | 73.2 (12.5) |  |
| Median (Q1, Q3) | 73.1 (63.5, 85.8) | 70.9 (59.0, 76.9) | 76.8 (63.8, 90.2) |  | 72.4 (66.0, 82.5) | 73.0 (65.6, 82.5) | 72.1 (66.2, 84.2) |  |
| VE/VCO₂ slope at RCP |  |  |  | 0.059 |  |  |  | 0.129 |
| Mean (SD) | 28.6 (4.7) | 30.0 (4.0) | 28.0 (4.9) |  | 29.6 (4.8) | 30.7 (5.3) | 28.4 (4.1) |  |
| Median (Q1, Q3) | 28.1 (25.4, 31.3) | 30.0 (26.9, 32.6) | 27.2 (25.0, 30.7) |  | 29.5 (25.2, 33.5) | 30.5 (26.5, 33.8) | 28.6 (25.0, 32.0) |  |
| Oues (ml/min/min) |  |  |  | 0.120 |  |  |  | 0.625 |
| Mean (SD) | 2,091.7 (548.8) | 2,189.3 (487.9) | 2,049.2 (571.8) |  | 2,194.4 (631.5) | 2,175.8 (684.1) | 2,212.4 (586.5) |  |
| Median (Q1, Q3) | 2,061.0 (1,715.0, 2,388.0) | 2,180.0 (1,812.0, 2,647.0) | 1,945.5 (1,677.0, 2,275.0) |  | 2,050.0 (1,664.0, 2,635.0) | 1,964.0 (1,657.0, 2,694.0) | 2,104.0 (1,738.0, 2,582.5) |  |
| rvedvi (ml/m2) |  |  |  | 0.146 |  |  |  | 0.052 |
| Mean (SD) | 123.6 (24.6) | 129.3 (18.1) | 121.0 (26.7) |  | 121.8 (24.3) | 127.8 (27.9) | 116.1 (19.1) |  |
| Median (Q1, Q3) | 123.5 (110.3, 136.2) | 124.3 (117.4, 142.3) | 122.8 (107.1, 135.9) |  | 119.3 (105.2, 139.8) | 129.3 (106.8, 141.4) | 113.5 (105.0, 127.1) |  |
| rvesvi (ml/m2) |  |  |  | 0.526 |  |  |  | 0.652 |
| Mean (SD) | 61.0 (16.8) | 61.2 (11.7) | 61.0 (18.7) |  | 57.1 (13.6) | 58.0 (15.2) | 56.3 (12.1) |  |
| Median (Q1, Q3) | 58.4 (51.0, 67.4) | 59.4 (54.4, 67.8) | 57.6 (48.1, 67.4) |  | 56.9 (46.6, 66.5) | 59.0 (46.2, 66.5) | 54.4 (46.8, 66.5) |  |
| rvef (%) |  |  |  | 0.185 |  |  |  | **0.026** |
| Mean (SD) | 51.5 (9.7) | 52.3 (5.0) | 51.1 (11.2) |  | 53.3 (5.6) | 54.7 (4.9) | 52.0 (5.8) |  |
| Median (Q1, Q3) | 51.0 (47.1, 55.0) | 51.4 (50.0, 55.0) | 50.8 (46.0, 55.0) |  | 53.5 (49.4, 56.3) | 54.3 (51.0, 58.3) | 51.0 (48.0, 55.7) |  |
| lvef (%) |  |  |  | 0.538 |  |  |  | **<0.001** |
| Mean (SD) | 56.8 (9.3) | 56.5 (5.0) | 57.0 (10.7) |  | 56.6 (4.6) | 58.6 (4.2) | 54.7 (4.2) |  |
| Median (Q1, Q3) | 56.0 (53.0, 58.1) | 56.0 (53.7, 57.8) | 55.6 (52.7, 58.5) |  | 56.1 (54.0, 59.0) | 58.0 (55.0, 62.3) | 55.0 (53.0, 57.0) |  |
| lvedvi (ml/m2) |  |  |  | 0.279 |  |  |  | 0.428 |
| Mean (SD) | 89.2 (16.6) | 86.2 (15.2) | 90.5 (17.2) |  | 81.2 (14.5) | 79.6 (14.1) | 82.6 (14.9) |  |
| Median (Q1, Q3) | 91.4 (78.0, 100.1) | 88.0 (72.8, 95.3) | 92.2 (80.3, 101.2) |  | 79.9 (73.1, 89.9) | 79.7 (72.5, 89.0) | 81.0 (73.3, 91.2) |  |
| lvesvi (ml/m2) |  |  |  | 0.551 |  |  |  | **0.020** |
| Mean (SD) | 39.6 (10.1) | 38.6 (8.1) | 40.1 (10.9) |  | 35.4 (8.3) | 33.0 (7.8) | 37.7 (8.3) |  |
| Median (Q1, Q3) | 39.8 (32.6, 46.3) | 38.5 (33.3, 42.8) | 40.1 (31.9, 46.9) |  | 34.7 (29.1, 39.9) | 31.4 (27.7, 38.2) | 37.4 (32.7, 41.2) |  |
| Pr (%) |  |  |  | **0.017** |  |  |  | 0.208 |
| Mean (SD) | 17.9 (16.7) | 23.7 (16.4) | 15.3 (16.3) |  | 25.9 (16.9) | 28.3 (17.0) | 23.6 (16.8) |  |
| Median (Q1, Q3) | 12.1 (5.1, 30.5) | 22.5 (7.0, 35.5) | 8.8 (4.5, 21.9) |  | 29.5 (6.4, 40.5) | 33.0 (12.3, 43.0) | 29.0 (4.8, 38.0) |  |
| ^1^n (%) | | | | | | | | |
| ^2^Wilcoxon rank sum test; Fisher's exact test; Wilcoxon rank sum exact test | | | | | | | | |

Supplementary Table 2: Baseline anthropometric, functional, and ventricular parameters in male patients stratified by exercise modality (bicycle vs treadmill) and age group (<18 years and ≥18 years). Legend: BMI = body mass index; IPAQ = International Physical Activity Questionnaire; CPET = cardiopulmonary exercise test; VO₂ = oxygen uptake; OUES = oxygen uptake efficiency slope; LVEDVI = left ventricular end-diastolic volume indexed to body surface area (BSA); LVESVI = left ventricular end-systolic volume indexed to BSA; LVEF = left ventricular ejection fraction; RVEDVI = right ventricular end-diastolic volume indexed to BSA; RVESVI = right ventricular end-systolic volume indexed to BSA; RVEF = right ventricular ejection fraction; PR = pulmonary regurgitation.
